# Paratesticular Serous Borderline Tumor in a Pediatric Patient

**DOI:** 10.1155/2020/8789143

**Published:** 2020-10-19

**Authors:** Itzel Araceli Ortiz Meza, Marco Antonio Ponce Camacho, Rodolfo Franco Márquez, Mauricio Delgado Morquecho, Raquel Garza Guajardo, Oralia Barboza Quintana

**Affiliations:** Department of Pathological Anatomy and Cytopathology, Universidad Autonoma de Nuevo Leon, Hospital Universitario ‘Dr José Eleuterio González, Monterrey, Nuevo León 64460, Mexico

## Abstract

Tumors of the paratesticular region are generally tumors of slow growth, with little symptomatology and, in most cases, benign in nature; in this area, a borderline serous tumor may arise hypothetically from Müllerian metaplasia of the tunica vaginalis, which is histologically identical to its ovarian counterpart. We present a 10-year-old male, with right gynecomastia and ipsilateral hydrocele, showing an enlarged right testicle with a volume of 12 ml and a left testicle with a volume of 10 ml. A right orchiectomy was performed, which presented a poorly defined tan tumor of 1.8 cm that occupied the vaginal and epididymal tunica, and infiltrates the testicular parenchyma. Histological sections revealed a cystic neoplasm, with hierarchical papillary projections, covered by one or several epithelial columnar and hobnail cells with moderate atypia and scant mitosis. Immunohistochemical reactions were performed, resulting positive for PAX-8, epithelial membrane antigen, and CK7, confirming the diagnosis of borderline serous tumor. Since the first reported case in 1986, few have been reported, the majority of these in adults with only three cases in children. In the few cases reported, the prognosis is usually favorable after surgical resection, with disease-free follow-up for up to 18 years.

## 1. Introduction

Tumors of the paratesticular region, which includes rete testis, epididymis, mesothelium and vestigial epithelium, and paratesticular soft tissues, are in general tumors of slow growth, with little symptomatology and, in most cases, benign in nature. Epithelial paratesticular tumors with Müllerian characteristics (serous, mucinous, clear cell, endometroid, and Brenner) are rare. The borderline serous tumor is analogous to its ovarian counterpart and was first reported by Young and Scully [1] in 1986, and afterwards, only few cases in adults have been reported, with three cases reported in children [2–4].

We present a case of a 10-year-old male patient with this unusual diagnosis.

## 2. Clinical Case

We present a 10-year-old male, son of nonconsanguineous parents, born at 36 weeks of gestation without complications, with adequate growth and development, who presented in 2018 gynecomastia and ipsilateral hydrocele. On the physical examination, both testicles were measured with the Prader orchidometer, showing an enlarged right testicle with a volume of 12 ml and a left testicle with a volume of 10 ml. Hormonal profile was performed, which reported normal results according to his age (testosterone 18.5 ng/dl; FSH 3.17mUI/ml, LH 0.33mUI/ml).

Pelvic ultrasound was performed with normal results, computed tomography of the chest reported gynecomastia, and abdominal computed tomography without abnormal findings. In addition, a karyotype of fifty metaphases was analyzed with normal results (46, XY).

**Figure 1:**
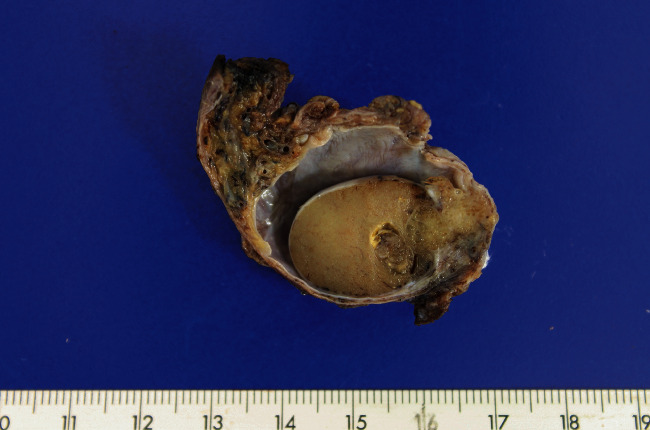
Right testicle. On cut surface shows a poorly defined 1.8 cm paratesticular tumor.

The patient underwent surgical correction of the hydrocele. During the procedure, a right paratesticular tumor was found, and orchiectomy was performed. The gross examination showed a testicle with 3.5 cm in diameter, and the cut surface presented a poorly defined tan tumor of 1.8 cm, occupying the vaginal tunica and epididymis and infiltrating the testicular parenchyma (Figure 1).

**Figure 2:**
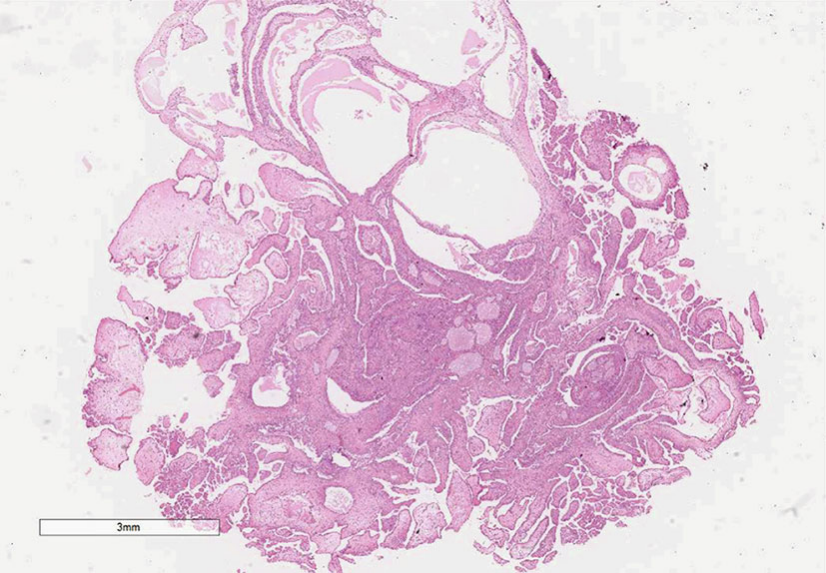
Cystic-looking tumor, with intermixed papillary projections.

**Figure 3:**
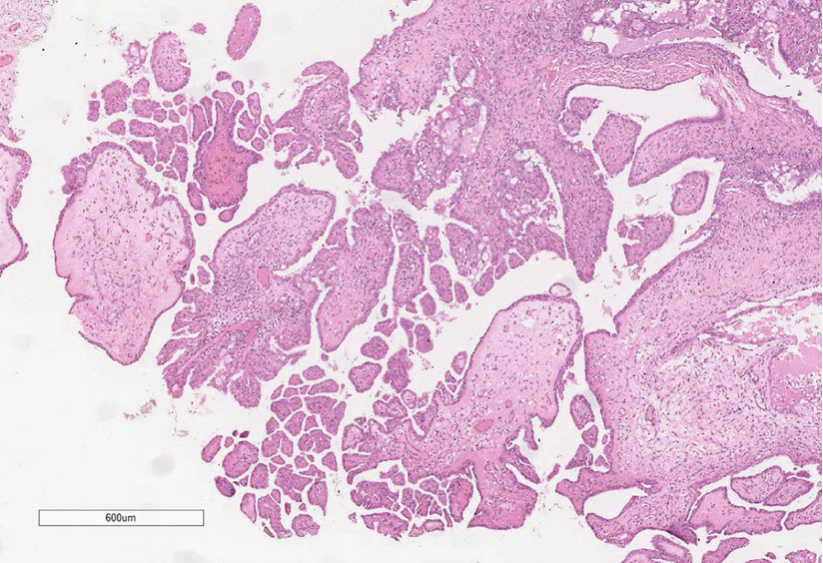
Hierarchical papillae with stroma of edematous appearance. 5x HyE.

**Figure 4:**
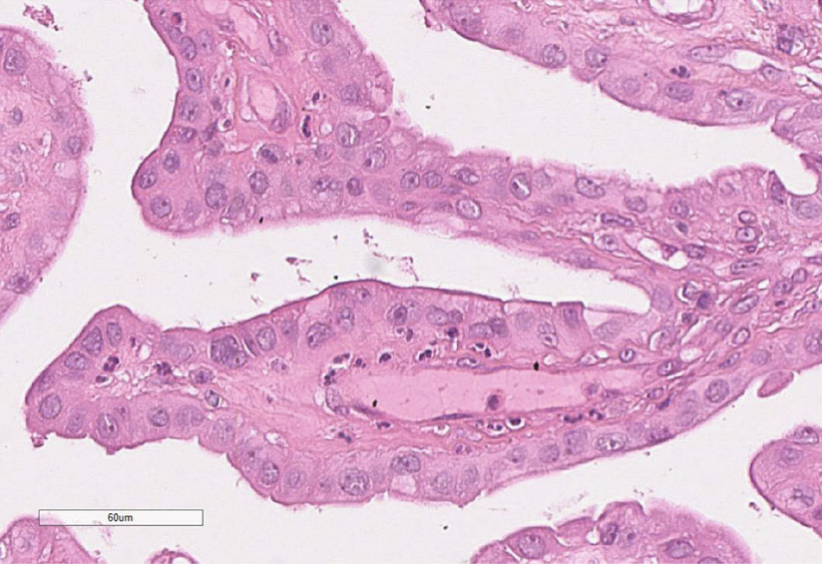
Papillae covered by several layers of columnar cells with moderate atypia. HyE 40x.

**Figure 5:**
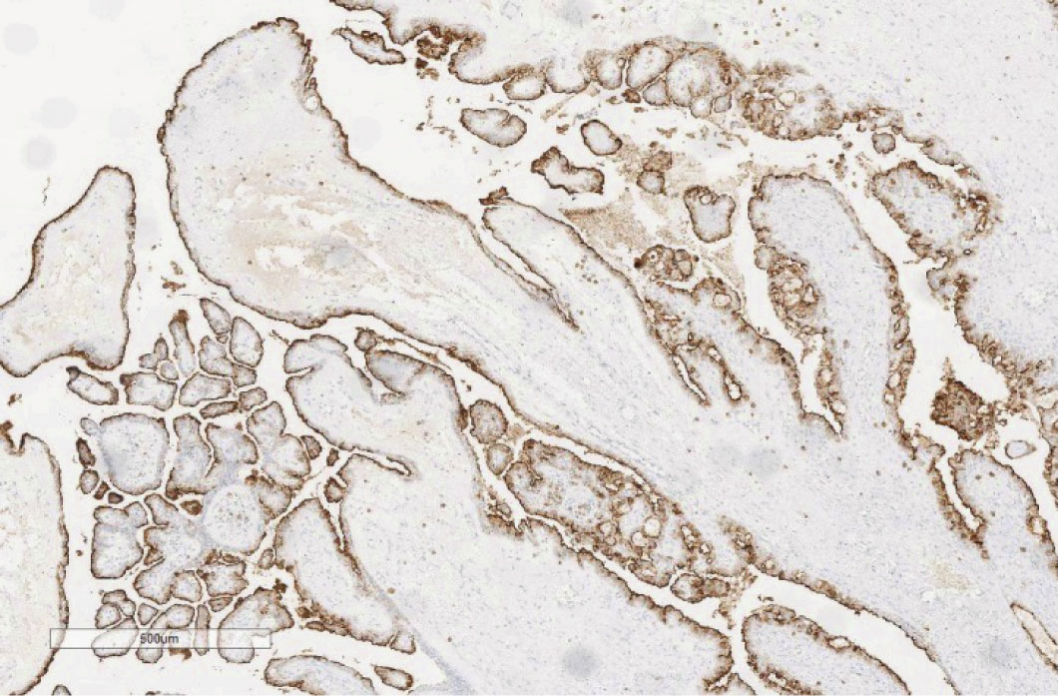
Epithelial membrane antigen.

**Figure 6:**
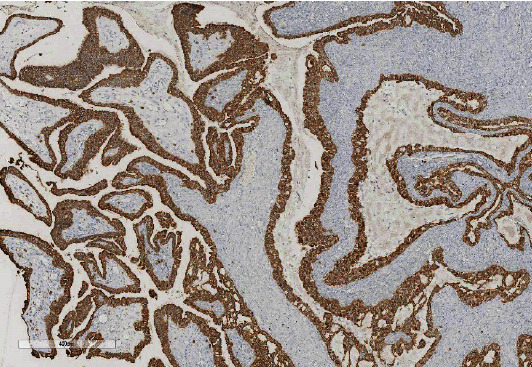
Cytokeratin 7.

**Figure 7:**
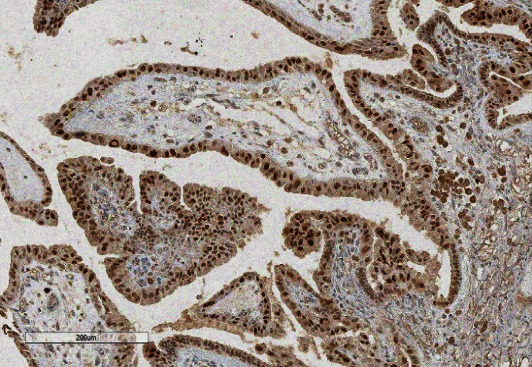
PAX8.

**Figure 8:**
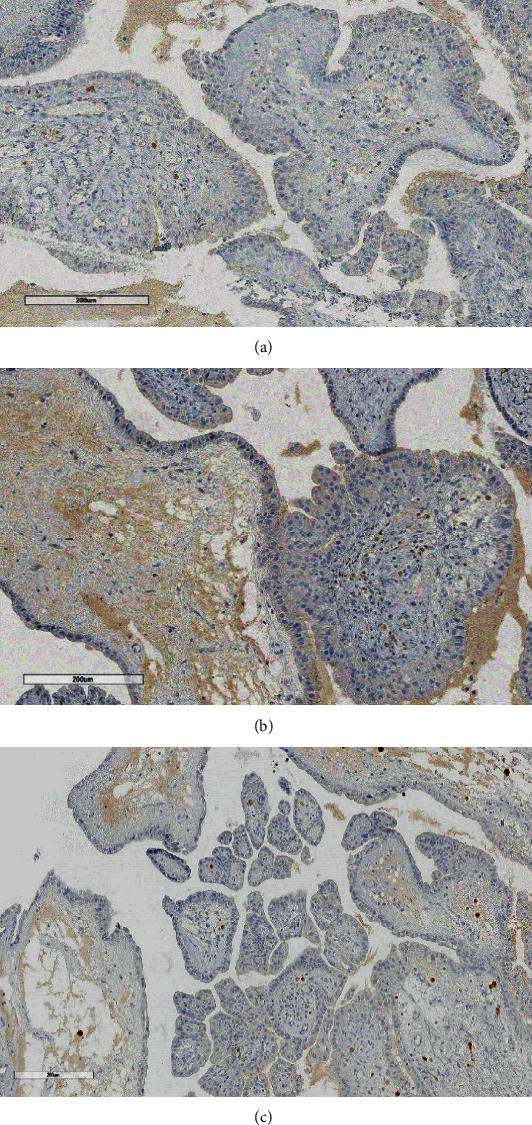
(a, b and c) The tumor was negative for calretinin, estrogen, and progesterone receptors, respectively.

In histological sections, a cystic neoplasm was observed, with hierarchical papillary projections, covered by one or several epithelial layers of columnar and hobnail cells with moderate atypia and scant mitosis (Figures 2–4). There was not any teratoumatous tissue in the background. Immunohistochemical reactions were performed, resulting PAX-8, epithelial membrane antigen (EMA), and cytokeratin 7 positive (Figures 5–7), confirming the diagnosis of borderline serous tumor. The tumor cells were negative for calretinin, estrogen, and progesterone receptors (Figure 8).

## 3. Discussion

Paratesticular neoplasms of Mullerian origin have morphological characteristics identical to ovarian Mullerian tumors. However, the anatomical origin is not well defined [2] In this area, a borderline serous tumor may arise hypothetically from Müllerian metaplasia of the tunica vaginalis, which is histologically identical to its ovarian counterpart; however, this theory is not fully accepted.

Due to the oddity of this neoplasm, little is known, not only of its origin but of its natural history. McClure et al. [2] presented a series of seven cases, which turns out to be the largest published series, where the average age corresponded to 56 years, with a case of a 14-year-old patient. Other pediatric cases have been reported by Walker et al. [3], a 14-year-old patient with borderline serous tumor of the tunica albuginea, with psammoma bodies, and Kleassen and coworkers [4], who presented a case of paratesticular borderline serous tumor in a patient aged 20 months with Proteus syndrome, whose association had not previously been studied as an etiological factor.

Patients are typically reported with painless masses in the paratesticular region. Most have hydrocele as an initial symptom. Serum markers are usually negative.

No specific imaging characteristics are recognized for borderline serous tumors of the paratesticular region [5]. Hsieh et al. [6] reported for the first time a paratesticular serous borderline tumor in a 59-year-old male correlating its magnetic resonance imaging findings, showing a pattern of mild hyperintensity in precontrast T1, with marked hyperintensity in T2, which is a pattern not associated with seminomatous or nonseminomatous germ cell tumors.

Macroscopically, a multicystic mass surrounded by a dense, pinkish, or whitish capsule can be observed. The average size ranges from 1 to 6 cm, with an average of 3.5 cm of diameter [2]. Located in tunica albuginea, or vaginal tunica, it has no association with rete testis or testicular involvement.

Histologically, it is a cystic-looking tumor, with papillary projections with intermingling papillary areas with abundant stroma and micropapillary portions, lined by one or several epithelial layers of columnar or hobnail cells alongside moderate atypia: scarce mitosis, with some psammoma bodies. It is usually positive for the following immunohistochemical markers: MUC-31, estrogen receptors, progesterone receptors, PAX-8, and cytokeratin 7. They have a low Ki67 of 1.3-10% (according to the McClure cohort) [2]. These tumors show negativity for cytokeratin 20, carcinoembryonic antigen, and calretinin. Supporting the hypothesis that these tumors are of Mullerian origin.

It is of vital importance to establish the differential diagnosis with other entities capable of being located in this area: (1) papillary cystadenoma, which is a benign tumor, the second in frequency in epididymis, usually asymptomatic, associated with Von Hippel Lindau syndrome in 68% of bilateral cases. Histologically, papillary projections in cystic spaces covered by cuboidal/columnar cells of single or double layer are observed, and the cells are clear or have eosinophilic cytoplasm, no mitosis, necrosis, or atypia are present; this histologically differentiates it from the borderline serous tumor (moderate atypia). They are usually positive for CK7, CK AE1/AE3, EMA, vimentin, PAX-8, and carcinoembryonic antigen. (2) Malignant mesothelioma which is a rare tumor in the paratesticular region (66 cases described in the literature) [7, 8]. Most occur between the 7th and 8th decades of life, but up to 10% have been described in children. These tumors are heterogeneous, with an epithelial component and a papillary component, presenting positivity for calretinin, vimentin, CK5, WT1, D240, and a high Ki67, unlike serous borderline tumor.

Most of the cases of paratesticular border line serous tumors were treated with orchiectomy, without proven metastases in the series in which there was follow-up, for up to 18 years [2]. Given the occasional elevation of CA-125 in the presentation, posttreatment monitoring may be appropriate in selected cases, but acceptance of this criterion remains controversial.

## 4. Conclusion

In conclusion, the borderline paratesticualr serous tumor is a rare tumor in the pediatric age, with apparently benign course; however, more experience with this type of neoplasm is needed to evaluate the biological behavior.
